# Effect of Fluoride Varnish on *Streptococcus mutans* Count in Saliva of Caries Free Children Using Dentocult SM Strip Mutans Test: A Randomized Controlled Triple Blind Study

**DOI:** 10.5005/jp-journals-10005-1001

**Published:** 2008-12-26

**Authors:** Deepti A, J Jeevarathan, MS Muthu, Rathna Prabhu V, Chamundeswari

**Affiliations:** 1 Lecturer, Department of Pediatric Dentistry, Meenakshi Ammal Dental College and Hospital, Chennai, India; 2 Lecturer, Department of Pediatric Dentistry, Meenakshi Ammal Dental College and Hospital, Chennai, India; 3 Professor, Department of Pediatric Dentistry, Meenakshi Ammal Dental College and Hospital, Chennai, India; 4 Professor and Head, Department of Pediatric Dentistry, Meenakshi Ammal Dental College and Hospital, Chennai, India; 5 Lecturer, Department of Microbiology, Meenakshi Ammal Dental College and Hospital, Chennai, India

**Keywords:** *Streptococcus mutans* count, Fluor Protector, Caries free children, Dentocult SM, Saliva.

## Abstract

*Aims:* The aim of this study was to estimate the count of *Streptococcus mutans* in saliva of caries free children using Dentocult SM strip mutans and to evaluate the effect of fluoride varnish on the *Streptococcus mutans* count in saliva of these caries free children.

*Methods and material:* Thirty caries free children were selected for the study based on the information obtained from a questionnaire prepared. They were randomly assigned into the control group and the study group consisting of ten and twenty children respectively. Samples of saliva were collected using the saliva strips from the Dentocult SM kit and after incubation the presence of the *Streptococcus mutans* was evaluated using the manufacturers’ chart. The study group was subjected to Fluor Protector fluoride varnish application after 24 hours following which the samples were collected again.

*Results:* The average *Streptococcus mutans* count in primary dentition of caries free children was in the range of 10^4^ to 10^5^ colony forming units/ml. The average *Streptococcus mutans* count in primary dentition of caries free children after Fluor Protector fluoride varnish application was below 10^4^ colony forming units/ml.

*Conclusion:* Fluor Protector fluoride varnish application showed a statistically significant reduction in the *Streptococcus mutans* count in saliva of the caries free children in the study group.

## INTRODUCTION

Dental caries is one of the most prevalent infectious diseases afflicting mankind.^1^ Dental caries has been traditionally described as a multifactorial disease that involves the interaction of various factors like host, agent, substrate and time. Most important in the understanding of caries process is that dental caries does not occur either in the absence of dental plaque or dietary fermentable carbohydrate, hence it is considered as a dietobacterial disease. *Streptococcus mutans* play a significant role in the development of dental caries and is the chief pathogen.^2^,^3^ Modern concepts consider caries as an interaction between genetic and environmental factors in which social, behavioral, psychological and biological factors are expressed in a highly complex interactive manner. If caries has to occur and progress the above conditions should be favorable.

A measure of caries activity and caries risk is the concentration of cariogenic bacteria within saliva. Although mutans streptococci (*Streptococcus mutans* and *Streptococcus sobrinus* in humans) and Lactobacilli are most commonly associated with dental caries development, several organisms have the ability to produce organic acids at levels that induce demineralization of tooth structure and lead to clinically detectable caries.

As dental caries is of multifactorial etiology preventive measures usually involve a combination of dietary counseling, oral hygiene measures and fluoride application. Caries activity is a compound diagnosis derived from immediate past experience, lesion progression and the clinical appearance of the lesion or cavities. Caries activity is evaluated on the basis of data obtained from clinical examination and assessment of factors associated with the pathogenesis of the disease. These data regarding dental caries can be collected by traditional visual inspection and probing or by some objective detection methods which rely on the mineral changes as a basis for evaluation of caries activity and risk assessment. None of these methods aim at the estimation of the chief pathogen *Streptococcus mutans*. Now microbial monitoring has been considered as an alternative method for evaluating current caries activity and future caries risk. Dentocult SM kit is a reliable method for measuring the status of dental caries in preschool children and also a valuable tool in the prevention and treatment of dental caries.^4^ Dentocult SM is one caries activity test which is helpful for the diagnosis of the presence of caries and prognosis of its progression based on the count of *Streptococcus mutans*.^5^

Fluoride works primarily via topical mechanisms including inhibition of demineralization, enhancement of remineralization at the crystal surfaces and inhibition of bacterial enzymes. Fluoride concentration as low as 0.02 to 0.06 ppm has been shown to enhance remineralization when enamel specimens were subjected to *in vitro* demineralization.^6^ Much of the research on fluoride focused on the interaction between it and the dental hard tissues with little or no attention paid to the effects of fluoride on the bacteria.

Fluoride at low concentration is bacteriostatic and at high concentration it is bactericidal.^7^ A high fluoride concentration in the oral cavity might inhibit acid production by bacteria and may reduce the number of certain species.^8^ Long-term use of fluoride mouth rinse might contribute to reduction of mutans streptococci.^9^ This present study was planned to evaluate the effect of fluor protector fluoride varnish on the *Streptococcus mutans* count in saliva of caries free children.

## MATERIALS AND METHODS

This study was carried out in the Department of Pedodontics and Preventive Dentistry in association with the Department of Microbiology, Meenakshi Ammal Dental College, Chennai.

### Sample Selection

All kindergarten children in Arulmigu Meenakshi Amman Matriculation Higher Secondary School, Chennai were screened by examiner (A) using mouth mirror and probe under day light. Forty eight caries free children with full set of primary dentition were selected. The parents of these children were asked to report to the department. The study was explained to the parents in detail. Child’s personal details, details of past medical history including any recent antibiotic exposure, past dental history including recent fluoride treatment, frequency of brushing, sweets/snacks intake and consumption of sugared/energy drinks and the brand of toothpaste to know about its fluoride content were obtained through a questionnaire from parents. Thirty subjects were selected for the study with the following inclusion criteria.

1. Caries free primary dentition
2. No history of antibiotics for the past 3-4 weeks
3. No history of fluoride treatment for the past 2 weeks

**Fig. 1:**
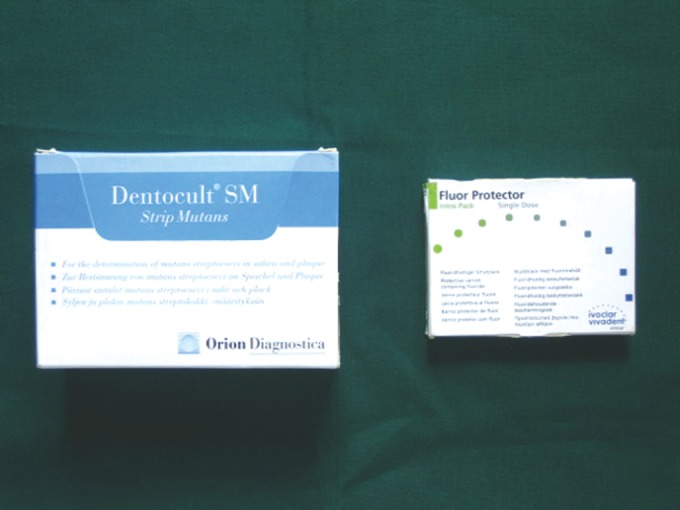
Dentocult SM kit and Fluor protector

Written consent was obtained from these parents. Each subject was assigned a specific number by asking them to pick up a lot which was done by examiner (B). The statistician randomized the numbers into the control group and the study group. Every subject had the equal chance of being in both the groups. Group-I (study group) consisted of 20 subjects and Group-II (control group) consisted of 10 subjects. The subjects were blinded about their group to which they belong. The materials used for this study were Dentocult SM kit (Orion Diagnostica, Finland) (Fig. 1) and Fluor Protector fluoride varnish (Vivadent, Germany) (Fig. 1).

### Collection of Saliva Sample

A fully equipped mobile dental van of our college was taken to the school for the collection of the saliva sample. The sample was collected 1-2 hours after eating/brushing as it could affect the growth of the bacteria. The Dentocult SM kit was removed from the igloo box and brought to room temperature before starting the procedure. The first step was to add the bacitracin discs to the selective culture vials using forceps, 15 minutes before collecting the saliva sample. The bacitracin tube was removed from the foil pack without removing the desiccant. After the desired number of discs were removed, the tube was reinserted, dispensing end first, into the foil pack. When taking the disc from a previously opened foil pack, the first two or three discs were discarded and the remaining discs were used.

**Fig. 2:**
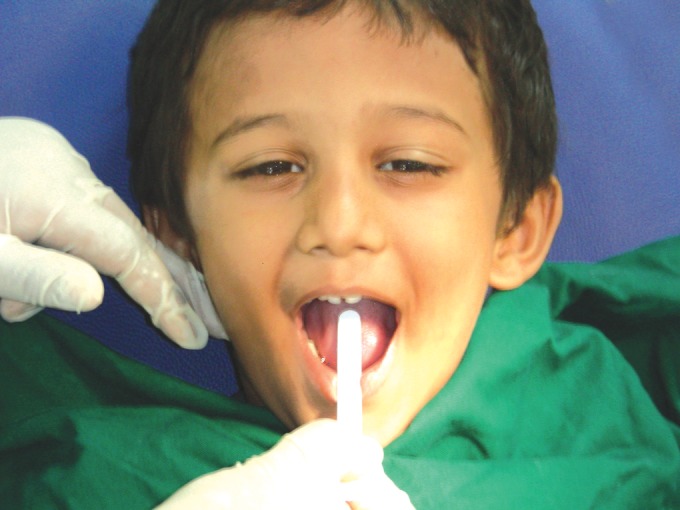
Sample collection using the strip

**Fig. 3:**
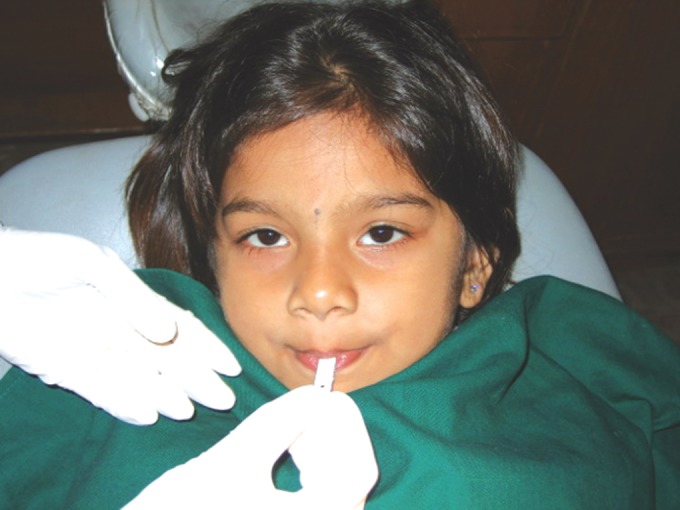
Removal of excess saliva from the strip

The round tipped saliva strips were removed from the pack. One saliva strip was used for each patient. The strips were rotated on the tongue ten times (Fig. 2), so that both sides of the strips are thoroughly inoculated by the subject’s flora. Excess saliva was removed by withdrawing the strip through closed lips (Fig. 3). The culture vials were shakento evenly distribute the bacitracin discs. The strips were then placed in the selective culture broth, with the smooth surfaces clipped and attached to the cap. The vials were then labeled as per their lot number and incubated in an upright position at 37°C for 48 hours with the cap opened one quarter of a turn. The minimum incubation time was 48 hours for the growth of the organisms. The results were interpreted by examiners A and C.

After the collection of the salivary sample from all the patients, fluoride varnish was applied to the subjects of study group by examiner (B) on the same day. First the tooth surfaces were completely dried with air syringe and then isolated with cotton rolls as per the manufacturer’s instructions. A high volume evacuator with saliva ejector and cheek retractor were also used. A thin layer of Fluor Protector fluoride varnish was applied on all the tooth surfaces using a suitable brush. The cotton rolls were removed after 1 minute and the patient was asked not to rinse the mouth immediately and not to eat or brush their teeth for 45 minutes. After 24 hours, saliva samples were again collected from the subjects of both the groups in the same van in the school. These were also incubated for the same time as before and the same interpreters evaluated the results again.

### Interpretation of Results

**Fig. 4:**
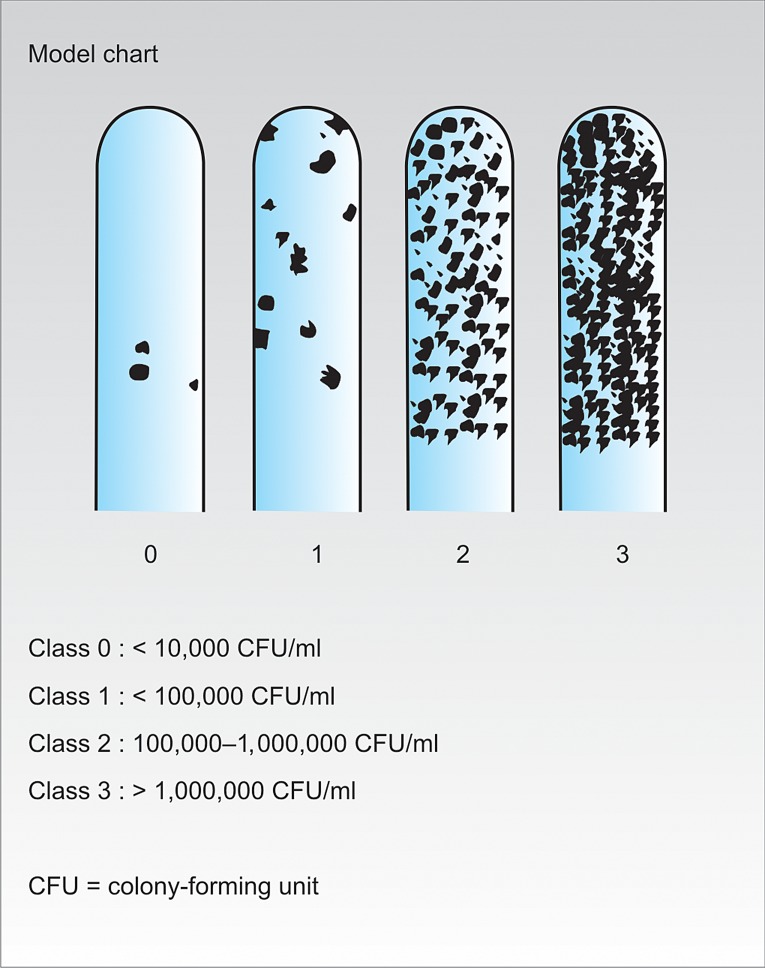
Manufacturer model chart

After incubation the presence of the *Streptococcus mutans* is confirmed by detecting light-blue to dark-blue, raised colonies on the inoculated surface of the strip. Colonies suspended in the culture broth were excluded from the evaluation. The results were evaluated according to the manufacturers’ chart (Fig. 4).

- Class 0: <10,000 CFU/ml (CFU- Colony forming unit)
- Class 1: <100,000 CFU /ml
- Class 2: 100,000-1000,000 CFU /ml
- Class 3: > 1000, 000 CFU/ ml

**Fig. 5:**
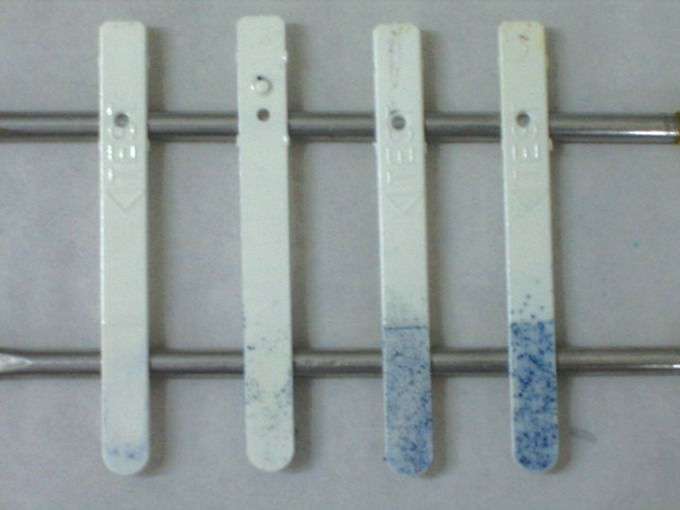
Different classes (0, 1, 2, 3) of *Streptococcus mutans* growth

The results were interpreted by two independent interpreters (examiner A and C), who were also blinded about the group division. Inspection was done with the growth sideways against a light or with the magnifying glass for raised colonies. The presence of epithelial cells on the strip surface should be differentiated from the mutans colonies. This was done by passing a gloved finger along the strip. The epithelial cells on the strip surface were smooth while the streptococci colonies were rough. Hence, only the rough colonies were accounted for the growth of the *Streptococcus mutans* (Fig. 5).

The subjects in the experiment group, the examiner who collected the saliva and the interpreter of the results were blinded about the division of groups.

## RESULTS

Table 1 shows the distribution of the subjects and their mean age in the study and the control group. There were 20 subjects in the study group and 10 in the control group. The mean age for the study group was 4.17 ± 0.80 (mean ± sd) and for the control it was 4.49 ± 1.12.

Table 2 shows the distribution of the various variables with pre-treatment bacterial count in the study group. All the subjects used only fluoridated toothpaste but the frequency of brushing, sweets/snacks intake and sugared drinks varied with the subjects of the study group. There was no statistically significant effect of the above variables on the pre-treatment bacterial count in the study group **(Chisquarep > 0.05)**.

**Table 1:** Table 1: Distribution of sample and mean age in years

*Sample*		*No. of subjects*		*Mean age (yrs)*
				*(mean ± sd)*
**Study Group**		20		4.17 ± 0.80
**Control Group**		10		4.49 ± 1.12

**Table 2:** TABLE 2: Comparison of descriptive variables of study group with pre-treatment bacterial score

*S.No.*		*Variables*		*Frequency of*		*Value*		*Bacterial score class*								*Total no.*		*p- value*
				*variables*				*0*		*1*		*2*		*3*		*of subjects*		*< 0.05 (sig)*
**1**		**Frequency of brushing**		One/day		1		0		14		5		0		19		0.117*(ns)
				Twice/day		2		0		0		1		0		1		
**2**		**Type of toothpaste**		Fluoridated		1		0		14		6		0		20		_
				Non-fluoridated		2		0		0		0		0		0		
**3**		**Frequency of sweets/snacks**		0/day		0		0		1		0		0		1		0.304*(ns)
				≤ 2/day		1		0		11		5		0		16		
				3-4/ day		2		0		2		0		0		2		
				> 4 / day		3		0		0		1		0		1		
**4**		**Frequency of sweet drinks**		0/day		0		0		0		0		0		0		0.117*(ns)
				≤ 2/day		1		0		14		5		0		19		
				3-4/ day		2		0		0		1		0		1		
				> 4 / day		3		0		0		0		0		0		

*Pearson chi-square test, sig–Significant, ns–Not significant

**Table 3:** TABLE 3: Comparison of descriptive variables of study group with post-treatment bacterial score

*S.No.*		*Variables*		*Frequency of*		*Value*		*Bacterial score class*								*Total no.*		*p- value*
				*variables*				*0*		*1*		*2*		*3*		*of subjects*		*< 0.05 (sig)*
**1**		**Frequency of brushing**		One/day		1		1		8		0		0		19		0.257*(ns)
								1											
				Twice/day		2		0		1		0		0		1		
**2**		**Type of toothpaste**		Fluoridated		1		1		9		0		0		20		_
								1											
				Non-fluoridated		2		0		0		0		0		0		
**3**		**Frequency of sweets/snacks**		0/day		0		1		0		0		0		1		0.279*(ns)
				≤ 2/day		1		8		8		0		0		16		
				3-4/ day		2		2		0		0		0		2		
				> 4 / day		3		0		1		0		0		1		
**4**		**Frequency of sweet drinks**		0/day		0		0		0		0		0		0		0.257*(ns)
				≤ 2/day		1		1		8		0		0		19		
								1											
				3-4/ day		2		0		1		0		0		1		
				> 4 / day		3		0		0		0		0		0		

*Pearson chi-square test, sig–Significant, ns–Not significant

**Table 4:** Table 4: Distribution of *Streptococcus mutans* score and average bacterial count for control group using strip mutans

*Bacterial score*		*Pre-treatment*				*Post-treatment*				p-value < 0.05 (sig)*
		*No. of*		*Average bacterial*		*No. of*		*Average bacterial*		
		*subjects*		*count*		*subjects*		*count*		
**Class 0**		1				2					
**Class 1**		7				7					
				10^4^ to 10^5^ **CFU/ml**				10^4^ to 10^5^ **CFU/ml**		**0.455 (ns)**
**Class 2**		1				0					
**Class 3**		1				1					

CFU/ml—Colony forming units/ml, *Mann—Whitney U test, sig—Significant, ns—Not significant

**Table 5:** TABLE 5: Distribution of *Streptococcus mutans* score and average bacterial count for study group using strip mutans

*Bacterial score*		*Pre-treatment*				*Post-treatment*				p-value < 0.05 (sig)*
		*No. of*		*Average bacterial*		*No. of*		*Average bacterial*		
		*subjects*		*count*		*subjects*		*count*		
**Class 0**		0				11					
**Class 1**		14				9					
				10^4^ to 10^5^ **CFU/ml**				<10^4^ **CFU/ml**		**0.000 (sig)**
**Class 2**		6				0					
**Class 3**		0				0					

CFU/ml—Colony forming units/ml, *Mann—Whitney U test, sig—Significant, ns—Not significant

Table 3 shows the distribution of the variables with the post-treatment bacterial count. Even though the variables varied with the subjects there was no statistically significant effect of them on the post-treatment bacterial count in the study group **(Chi-square p > 0.05)**.

Table 4 shows the distribution and average pre and posttreatment bacterial count in the control group. There was no statistically significant difference in the distribution of bacterial score in the control group **(Mann-Whitney U test p= 0.455)**. The average pre and post-treatment bacterial count was in the range of 10^4^ to 10^5^ CFU/ml.

Table 5 shows the distribution and average pre and posttreatment bacterial count in the study group. There was a statistically significant difference in the bacterial count **(Mann-Whitney U test p = 0.000)**. The average pretreatment bacterial count was about 10^4^ to 10^5^ CFU/ml where as it was less than 10^4^ CFU/ml after fluoride varnish application.

**Table 6:** Table 6: Inter-examiner (A and C) relationship between pre-treatment and post-treatment scores

*Inter-examiner*		*No. of*		*Pre-*		*Post-*
*relationship (kappa)*		*subjects*		*treatment*		*treatment*
		30		0.926		0.821

Table 6 shows the inter-examiner relationship between the pre-treatment and post-treatment bacterial count in all the subjects. The value was **0.926** and **0.821** using **Cohen’s kappa.**

Table 7 shows the distribution of the pre-treatment bacterial count in the plaque of caries free primary dentition. The average number of bacteria can be in the range of 10^4^- 10^5^ colony forming units/ml. The mean and the standard deviation for bacterial score were about 1.33 ± 0.55.

**Table 7:** Table 7: Distribution and average of bacterial scores in caries free children

*Bacterial score*		*No. of bacteria (CFU/ml) colony forming units/ml*		*No. of subjects*		*Median*		*Mean ± sd*
**Class 0**		< 10^4^		1		10^4^-10^5^		1.33 ± 0.55
**Class 1**		10^4^-10^5^		21				
**Class 2**		10^5^-10^6^		7				
**Class 1**		> 10^6^		21				

## DISCUSSION

Saliva is well adapted to protect the teeth against dental caries. Saliva’s buffering capacity; the ability of the saliva to wash the tooth surface, to clear bacteria, and to control demineralization and remineralization and saliva’s antibacterial properties; all contribute to its essential role in the health of teeth. The importance of saliva as buffer depends largely on it ability to control the reductions in pH resulting from bacterial action on metabolic substrates found in dental plaque. Bicarbonate is the major buffer in saliva, and its concentration in saliva increases as salivary flow rate increases. Other buffers present in saliva include phosphates, urea and arginine-rich proteins.

In addition to the ability of saliva to act as a lavage vehicle and to provide buffering of acid on the tooth surface, individual components of saliva have been shown to have effects on bacterial activity or on demineralization and remineralization of the tooth structures. These components are lactoferrin, lysozyme, statherin and proline rich proteins, histatins, mucins, cystatins, peroxidase and immunoglobulins.

Fluoride has been found to be the most effective cariostatic agent in the field of dentistry especially in pediatric dentistry. In the past few decades it has completely changed the approach of treatment from a therapeutic concept to a more preventive approach. The action of fluoride for caries prevention are multiple such as effects on the teeth, bacteria and plaque. In the teeth it alters the physiochemical properties by making it more resistant to acid dissolution due to formation of fluoroapatite or fluorohydroxyapatite. It also increases the posteruptive maturation, enhances remineralization and inhibits demineralization. In the bacteria it inhibits various enzymes like enolases, phosphatases, proton extruding ATPases and pyrophosphatases.^8^ Hence, in our study the effect of Fluor Protector fluoride varnish on *Streptococcus mutans* count in saliva of caries free children was analyzed using Dentocult SM strips.

Subjects (30) with only caries free dentition were chosen for our study to assess the actual effect of fluoride varnish on the *Streptococcus mutans* count in saliva. These subjects were selected based on the information obtained through the questionnaires filled by the parent. This also had the variable data’s regarding the type of toothpaste, frequency of brushing, sweets/snacks intake and sugared/energy drinks. These data were collected in order to know the diet pattern of subjects and to evaluate their role on Streptococcus mutans count in saliva. There was no statistical significance between the pre-treatment bacterial score and frequency of brushing, sweets/snacks intake and sugared/energy drinks consumption.

Yoshiara A et al^9^ compared variables like sealants, dfs, DMFS, frequency of sweet drinks, sweets/snacks, and brushing, fluoridated and non-fluoridated paste with the bacterial count while evaluating the effect of fluoride mouthrinse on it. They did not find any significant effect of fluoridated tooth paste on the *Streptococcus mutans* count. But only the frequency of sweets/snacks, dfs and sealants had significant effect on bacterial count. The reason for the significance could be the larger sample size of their study and moreover, the bacterial score was also grouped as high (class 2 and 3) and low (class 1 and 2). Hence, with the above dependant variables logistic multiple regressions was carried out to find the actual effect of fluoride mouth rinse on *Streptococcus mutans* count. But in our study as there was no statistical significant difference found, no such analysis was done. All the subjects of the study group used only fluoridated tooth paste and hence no statistical analysis was done to evaluate its effect on bacterial count. Moreover, the subjects of the control group also used fluoridated paste. In another study no difference in the level of mutans streptococci between subjects using or not using fluoridated tooth paste was found.^10^

Saliva samples were collected with the aim of assessing the overall effect of fluoride varnish on *Streptococcus mutans* count. Dentocult SM is the best test for the diagnosis of the presence of caries and prognosis of it with high statistical significance.^4^,^5^ Dentocult SM is better than Dentocult LB for caries risk assessment. Moreover, this test is chair side, more patient compliance especially for young age, minimal armamentarium needed, less time consuming and easy sample collection. Davenport ES et al^11^ compared Dentocult SM kit with conventional method and found these dip-slide tests provide a simple and suitable method of screening salivary *Streptococcus mutans* level, which may have a useful role in caries risk assessment. In a similar study, the sensitivity, specificity and accuracy of Dentocult SM were better than the conventional methods.^12^

Fluor Protector fluoride varnish was applied after the collection of pre-treatment saliva sample. Twenty four hours later, post-treatment saliva samples were again collected as before to check and compare the bacterial count. There was no statistically significant difference between the posttreatment bacterial score and frequency of brushing, sweets/ snacks intake and sugared/energy drinks consumption. According to Ekenbach SB et al^13^ who studied the colonization of cariogenic bacteria in plaque of exposed root surfaces after application of four different varnishes found no statistically significant difference between baseline and over time (1 week, 1 month and 6 months) samples with Fluor Protector. Hence a twenty four hour sample was collected after which fluoride varnish was applied even to the control group. This was done so that they are not deprived of the preventive effect of fluoride varnish. This study was triple blinded as explained in materials and methods to eliminate bias and to get more authenticated results.

The average pre and post-treatment bacterial count of the control group is in the range of 10^4^-10^5^ CFU/ml. The average pre-treatment bacterial count of the study group was in the range of 10^4^-10^5^ CFU/ml where as the posttreatment count is only less than 10^4^ CFU/ml. Twetman et al^14^ studied the fluoride concentration in whole saliva and in separate gland secretions after a single application of three different fluoride varnishes with contrasting levels of fluoride in a randomized crossover design. In whole saliva, the fluoride levels were significantly elevated one hour after the varnish applications compared with baseline. Similar patterns were unveiled in the parotid and submandibular-sublingual secretions, although the increase in fluoride concentration was modest. The elevated levels did not exceed six hours for any of the varnish tested. The results of this study suggest a correlation between the concentration of fluoride of the varnish and fluoride levels obtained in saliva after application.

In an *in vitro* study by Munshi AK et al^15^ where they compared the antibacterial effect of Bifluorid 12, Fluoritop- SR and Fluor Protector in Blood agar and MSBA (Mitis Salivarius Bacitracin Agar), it was found that Fluor Protector had the least inhibitory effect. This was due to the low fluoride content of Fluor Protector when compared to other fluoride varnishes. Even though Fluor Protector has low concentration of fluoride and caries inhibiting activity when compared to Duraphat, the fluoride deposited in teeth was more in Fluor Protector than Duraphat.^15^

This fluoride which could have leached out from the teeth would have inhibited the growth of bacteria. The long term use of fluoride mouth rinses had significant antibacterial action on the *Streptococcus mutans*.^9^

Thus an average number of colony forming units in caries free children with primary dentition could be about 10^4^ to 10^6^ CFU/ml. Levels accepted as risk for caries in adults and older children per ml of stimulated saliva was about 10^5^ to 10^6^ CFU/ml. The conclusions derived from the results of this study were

- Fluor Protector fluoride varnish has a statistically significant reduction in the *Streptococcus mutans* count in saliva after 24 hours.
- The average *Streptococcus mutans* count in primary dentition of caries free children was in the range of 10^4^ to 10^5^ colony forming units/ml.
- The average *Streptococcus mutans* count in primary dentition of caries free children after Fluor Protector fluoride varnish application was below 10^4^ colony forming units/ml.

As this was the first study in caries free children, further studies with larger sample size and a placebo group can reveal the long term effect of Fluor Protector fluoride varnish on the *Streptococcus mutans* count in saliva of carious and caries free dentition.
